# The Quartet Data Portal: integration of community-wide resources for multiomics quality control

**DOI:** 10.1186/s13059-023-03091-9

**Published:** 2023-10-26

**Authors:** Jingcheng Yang, Yaqing Liu, Jun Shang, Qiaochu Chen, Qingwang Chen, Luyao Ren, Naixin Zhang, Ying Yu, Zhihui Li, Yueqiang Song, Shengpeng Yang, Andreas Scherer, Weida Tong, Huixiao Hong, Wenming Xiao, Leming Shi, Yuanting Zheng

**Affiliations:** 1 State Key Laboratory of Genetic Engineering, School of Life Sciences, Human Phenome Institute and Shanghai Cancer Center, Fudan University, Shanghai, China; 2 Greater Bay Area Institute of Precision Medicine, Guangzhou, Guangdong China; 3 Intelligent Storage, Alibaba Cloud, Alibaba Group, Hangzhou, Zhejiang China; 4 Institute for Molecular Medicine Finland (FIMM), University of Helsinki, Helsinki, Finland; 5 EATRIS ERIC-European Infrastructure for Translational Medicine, Amsterdam, the Netherlands; 6 Division of Bioinformatics and Biostatistics, National Center for Toxicological Research, US Food and Drug Administration, Jefferson, AR USA; 7 Office of Oncological Diseases, Office of New Drugs, Center for Drug Evaluation and Research, US Food and Drug Administration, Silver Spring, MD USA; 8 International Human Phenome Institutes (Shanghai), Shanghai, China

**Keywords:** Quartet Data Portal, Multiomics, Quartet Project, Quality control, Reference materials, Reference datasets, Performance evaluation, Reproducibility, Evolving technologies, Interactive visualization

## Abstract

The Quartet Data Portal facilitates community access to well-characterized reference materials, reference datasets, and related resources established based on a family of four individuals with identical twins from the Quartet Project. Users can request DNA, RNA, protein, and metabolite reference materials, as well as datasets generated across omics, platforms, labs, protocols, and batches. Reproducible analysis tools allow for objective performance assessment of user-submitted data, while interactive visualization tools support rapid exploration of reference datasets. A closed-loop “distribution-collection-evaluation-integration” workflow enables updates and integration of community-contributed multiomics data. Ultimately, this portal helps promote the advancement of reference datasets and multiomics quality control.

**Supplementary Information:**

The online version contains supplementary material available at 10.1186/s13059-023-03091-9.

## Background

Reference materials, which are sufficiently homogeneous and stable with respect to one or more specified properties, play a crucial role in enhancing the reliability of multiomics profiling [1, 2]. These materials, characterized with omics reference datasets (also known as benchmarks), serve as standards for instrument calibration, and for evaluating the performance of omics measurement and computational methods. The utilization of reference materials is accompanied by a comprehensive suite of tools, encompassing standardized analytical pipelines and quality control tools for performance assessment of each omics profiling [3–8]. In addition, the publicly accessible datasets derived from different platforms, protocols, laboratories, and batches contribute to the richness of available resources for omics quality control.

Despite the development of various reference materials, efforts to enhance their public accessibility through a user-friendly data portal are still nascent [2, 9, 10]. Several official websites, such as those proposed by GIAB [11, 12], HUPO-PSI [13], MAQC/SEQC [7, 14–16], mQACC [17, 18], and Sequin [19–21], provide users with access to reference materials, pre-existing data or tools (Table 1). They have not fully met the needs of users seeking an integrated workflow from sample acquisition to data quality assessment. For example, the GIAB website (https://www.nist.gov/programs-projects/genome-bottle) covers a wide range of features, which is accomplished by linking to multiple external sources, including the NIST store, nearly ten data storage paths, and tools developed by the GA4GH benchmarking team to evaluate the performance of germline variant callers [5]. However, users are still unable to gain a comprehensive overview of datasets from different sources, free access to reference materials, and online assessments. In this context, a comprehensive solution that integrates sample acquisition, data download and upload, and online analysis into one platform would benefit users with more convenient follow-up processes after accessing reference materials.

**Table 1:** Data portals for quality control studies based on reference materials

	**GIAB**	**HUPO-PSI**	**MAQC/SEQC**	**mQACC**	**Quartet**	**Sequin**
Reference materials request	Links to NIST, Coriell, and PGP	No	No	No	Direct application	Direct application
Data availability	Links to FTP	No	Links to NCBI SRA and FTP	No	Direct download	No
Analysis tools	A package of hap.py and a web-based implementation of this tool in GA4GH Benchmarking app on precisionFDA	Dozens of packages for exchanging data formats, capturing molecular interactions, and reporting data quality	Example codes for benchmarking, a R package containing summarized read counts and exon-exon junctions	No	Online applications for analyzing and reporting the user-submit data quality	No
Data retrieval	No	No	No	No	Raw sequencing data or quantitative profiles, and structured metadata	No
Account requirement	Yes	No	No	No	Yes	Yes
Additional assistance	Connections to opportunities, e.g., workshops and collaborations	Controlled vocabularies, reporting guidelines for proteomics	Many types of tutorials	No	Interactive visualization	No

Integrating continuous feedback from the community on the utilization of reference materials could furnish a data foundation for accelerating the development of existing quality control system. Intra- and inter-lab performance varies considerably in terms of instruments, experimental protocols, and computational pipelines [14, 22]. Exploring these differences is of utmost importance in quality control investigations, but cannot be comprehensively addressed within a single study. Therefore, it is essential to integrate real-world data from communities with complementary technical strengths and complex performance. A paradigm model is the crowdsourced precisionFDA challenges, which leverages the power of community participants to identify the QC tools with high accuracy and robustness [23], and to upgrade benchmarks for easy- and difficult-to-map genomics regions [24], etc. This exemplary model deserves to be extended to more dimensions with other types of omics studies to help researchers gain the knowledge and resources to ensure data quality and thus improve the reliability of omics-based biological discoveries.

In this context, we developed the Quartet Data Portal around the Quartet Project. The Quartet Project (https://chinese-quartet.org) was launched for quality control of multiomics profiling based on the large quantities of multiomics reference materials derived from immortalized B-lymphoblastoid cell lines of a monozygotic twin family. See accompanying papers on the overall project findings [25], genomics [26], transcriptomics [27], proteomics [28], metabolomics [29], and batch-effect monitoring and correction [30] with the Quartet multiomics reference materials. With the community-wide efforts, extensive datasets across platforms, labs, protocols, and batches were generated for the multiomics characterization of the reference materials. The Quartet Project team has developed the corresponding reference datasets, QC metrics, and analysis tools for genomics, transcriptomics, proteomics, and metabolomics to accompany the reference materials, resulting in a comprehensive quality control system. The Quartet Data Portal is a central hub that integrates all these resources and is dedicated to promoting the use of reference materials and to continuously upgrade the Quartet quality control system. Functions provided include channels for requesting multiomics reference materials, tools for obtaining multi-level data, interactive visualization for exploring reference datasets, and online applications for quality assessing of user-submitted data. The portal is compliant with the FAIR (Findability, Accessibility, Interoperability, and Reusability) principles and is aimed for advancing scientific data management and community sharing efforts [31].

## Results

### Overview of the Quartet Data Portal

**Fig. 1:**
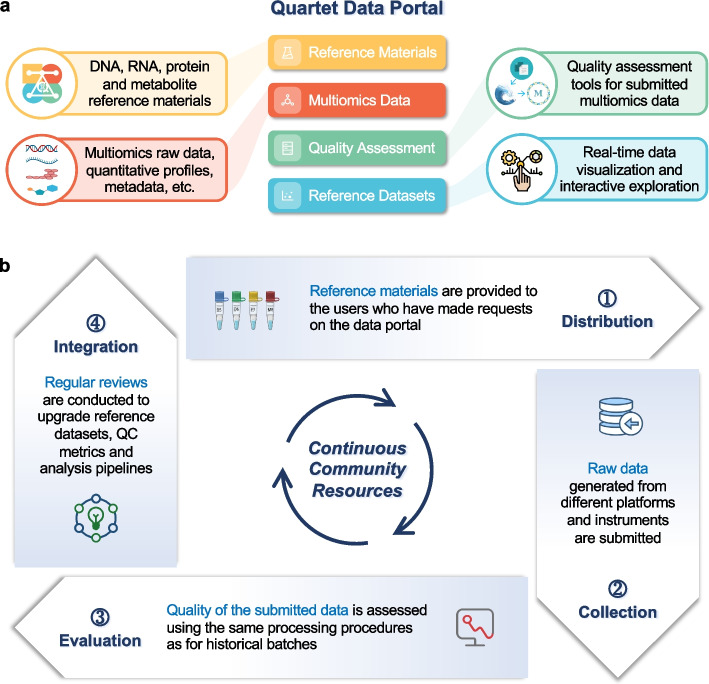
Overview of the Quartet Data Portal. **a** Reference materials, multiomics data, quality assessment, and reference datasets constitute the core of the Quartet Data Portal, a user-friendly web-server for requesting samples, accessing to multiomics data, evaluating quality of submitted data, and visualizing reference datasets. **b** The Quartet Data Portal uses a “distribution-collection-evaluation-integration” closed-loop workflow. Continuous requests for reference materials by the community will generate large amounts of data from the Quartet reference samples under different platforms and labs. This helps improve the quality control metrics and analysis pipelines, as well as to gradually improve the reliability and application scope of the reference materials and datasets

The Quartet Data Portal serves as a comprehensive platform for integrating the diverse resources of the Quartet Project as depicted in Fig. 1a. This portal encompasses four key modules, each offering unique functionalities: (1) Reference materials: A unique online channel is provided for the public to request reference materials. Essential information on reference DNA, RNA, proteins, and metabolites is displayed in this module. (2) Multiomics data: A data hub for accessing multi-level omics data, which involves metadata, raw datasets, intermediate datasets, and profiles. (3) Quality assessment: Reproducible analysis tools are developed to assess the quality of user-submitted data and to generate quality assessment reports. (4) Reference datasets: This module contains the reference datasets of high-confidence small variants (SNVs and Indels), structural variants (SVs), RNAs, proteins, and metabolites, as well as interactive visualization tools for quick understanding and exploration.

### The “distribution-collection-evaluation-integration” model supports the continuous evolution of technologies

Users who have applied for the reference materials are encouraged to upload raw data from sequencing or mass spectrometry back to the Quartet platform for analysis. At this point, the Quartet Data Portal enables an effective interaction with the community, forming a closed-loop “distribution-collection-evaluation-integration” workflow (Fig. 1b), which benefits both users and the Quartet platform. Through this workflow, users gain access to a comprehensive quality assessment report and all relevant intermediate data generated during the analysis process. This enables them to obtain valuable insights into the quality and characteristics of their data. Simultaneously, the Quartet team can further improve the overall quality control system by leveraging newly submitted data with a stringent process of review and integration.

The data collected by the Quartet Data Portal from the research community is diverse and encompasses various types of information. These data sources are continually expanding and curated, ensuring the inclusion of multi-level data from genomics, transcriptomics, proteomics, and metabolomics. They are generated across different platforms, laboratories, protocols, and experimental batches, capturing the breadth and diversity of real-world research endeavors. On this basis, the Quartet Data Portal regularly updates its multiomics reference datasets. These reference datasets serve as “ground truth” for assessing the quality of user-submitted data. They provide a valuable resource for researchers to compare and evaluate their own data against established reference datasets. In addition, the Quartet Data Portal also offers version-controlled data analysis pipelines. These pipelines are aligned with those used to build the reference datasets and are continually updated and refined as the reference datasets are upgraded. By employing these pipelines, researchers can assess the quality and reliability of their multiomics data, further enhancing the overall research quality and reproducibility.

### Multiomics reference materials and multi-level data resources are accessible

**Fig. 2:**
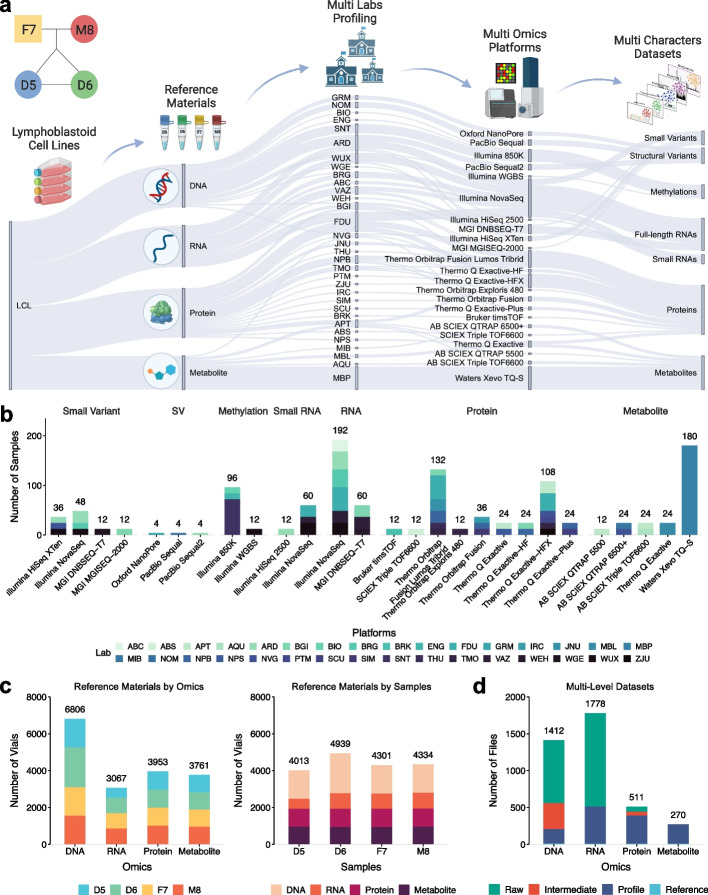
Available reference materials and data resources. **a** The Sankey diagram displays a general view of the Quartet Project supported by the Quartet Data Portal. **b** Bar plots of the number of samples used to generated multiomics datasets in 32 labs with 24 platforms. The multiomics features include small variants, structural variants (SV), methylations, small RNAs, full-length RNAs, proteins, and metabolites. **c** Bar plots of the number of available Quartet multiomics reference materials by omics and samples. **d** Bar plots of the number of files for raw datasets (level 1), intermediate datasets (level 2), profiles (level 3), and reference datasets (level 4)

The resources of the Quartet Data Portal cover the whole process of multiomics data generation and data analysis in the Quartet Project (Fig. 2a). The Quartet reference materials are extracted from immortalized B-lymphoblastoid cell lines (LCLs), which were established from the peripheral blood samples of four members of a family Quartet including father (F7), mother (M8), and monozygotic twin daughters (D5 and D6). Aliquots of DNA, RNA, proteins, and metabolites from the same lot were randomly distributed to different labs. Except for the long-reads sequencing platforms, the reference materials were profiled within a batch in a site in three replicates for each of the four samples (donors). For long-read sequencing, one replicate for each reference material was sequenced. Each batch of samples distributed was blinded to avoid specific experimental sequences affecting the objective assessment of lab proficiency. Finally, large quantities of data across six omics types generated from 24 platforms at 32 labs are collected and available via the Quartet Data Portal.

Specifically, 108 DNA samples were subjected to four short-read sequencing platforms (Illumina HiSeq and NovaSeq, MGI MGISEQ-2000, and DNBSEQ-T7) at six labs for the characterization of small variants. Additionally, 12 DNA samples were measured on three long-read (Oxford Nanopore Technologies (ONT), Pacific Biosciences (PacBio) Sequel and Sequel II) sequencing platforms at three labs to investigate SVs. Epigenomic (methylomic) data, involving 108 DNA samples, was obtained through Illumina EPIC (850 K) array and whole-genome bisulfite sequencing (WGBS) at four labs. RNA sequencing data of 252 samples were generated on MGI DNBSEQ-T7 and Illumina NovaSeq using poly(A) selection or RiboZero library preparation protocols at eight labs. Small RNA sequencing data of 72 samples were generated on Illumina NovaSeq and HiSeq 2500 at four labs. Proteins (annotated from peptides) of 384 samples were measured on nine LC–MS/MS-based proteomics platforms (Thermo Scientific Q Exactive, Q Exactive-HF, Q Exactive-HFX, Q Exactive-Plus, Orbitrap Fusion Lumos Tribrid, Orbitrap Fusion, Orbitrap Exploris 480, Bruker timsTOF, and SCIEX Triple TOF6600) at 16 labs. Metabolites of 264 samples were measured on five LC–MS/MS-based metabolomics platforms (Thermo Scientific Q Exactive, SCIEX Triple TOF6600, QTRAP 6500 + , QTRAP 5500, and Xevo TQ-S) at six labs (Fig. 2b).

To date, tens of thousands of vials of reference DNA (10 μg/vial), RNA (5 μg/vial), proteins (10 μg of dried and tryptic peptide mixtures/vial) and metabolites (dried cell extracts from 10^6^ cells/vial) that have already been verified for homogeneity and stability are stored in the − 80 °C freezers (Fig. 2c). More than 40 TB of 3917 multi-level data files including raw data (level 1), intermediate data (level 2), profiles (level 3), and reference datasets (level 4) have been managed hierarchically in the Quartet Data Portal (Fig. 2d). All levels of genomic data are available; intermediate files (Binary Alignment Map files) are not retained for transcriptomic data; and for metabolomics, only profiles and reference datasets are provided. In addition, metadata involved in the whole process, from study design to the final step of data analysis, are documented and available. In the first release, a total of 5,373,058 small variants, 19,129 SVs, 15,372 full-length mRNAs, 3197 proteins, and 82 metabolites are contained in the reference datasets.

### Permissions policy of the Quartet Data Portal

#### Reference materials request

**Fig. 3:**
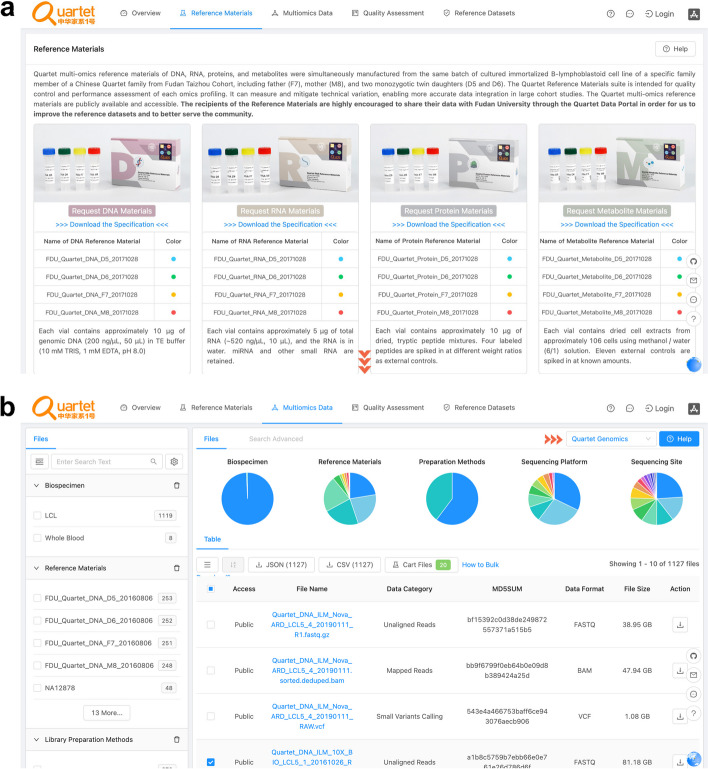
Interfaces for requesting reference materials and reference datasets from the Quartet Data Portal. **a** Interface for requesting Quartet multiomics reference materials. **b** Interface for requesting Quartet multiomics datasets

Users can obtain the characteristics of the reference materials and apply for the samples through an online channel (Fig. 3a). Access to Quartet reference materials is free of charge for scientific research use, subject to sharing the data with the Quartet team and ensuring future public access through platforms such as GSA and SRA. Compliance with the regulations set by the Human Genetic Resources Authority of China (HGRAC) is also mandatory. Further details please refer to https://docs.chinese-quartet.org/policies/reference_materials_policy/.

#### Data request

The multiomics datasets released on the Quartet Data Portal are open-access, with approval by HGRAC. Multi-level datasets and structured metadata can be obtained in a data hub which provides the browser, search, and download functions. As shown in the Pie charts of files that meet the filtering conditions and search criteria at the same time (Fig. 3b), a faceted search interface allows the filtration according to omics type, data category, data format, platform, protocol, etc. Retrieved datasets and corresponding metadata can be downloaded in flat files. Further details please refer to https://docs.chinese-quartet.org/policies/data_request_policy/.

#### Account registration

An account will be granted after a successful request for reference materials through the Quartet Data Portal. For users obtaining reference materials outside the portal, a request for an account should be made to quartet@fudan.edu.cn. With an account, the user can benefit from the free online quality assessment. For further details, please refer to https://docs.chinese-quartet.org/policies/account_registration_policy/.

#### Data submission

Applicants for Quartet reference materials must provide essential metadata and omics data to the Quartet team, which could be used for periodically upgrading the reference datasets. Metadata is structured across the whole experimental process and designed according to the NCI Thesaurus standard [32]. Users need to prepare data according to the requirements of each omics type, and the format and integrity will be checked. Data contributors have a private access period of three months to manage their data, after which the Quartet team informs them about upcoming data deletion. For further details please refer to https://docs.chinese-quartet.org/policies/data_submission_policy/.

### Interactive visualization enables instant exploration of the reference datasets

Understanding the characteristics of the reference datasets is essential but needs to be made relatively intuitive for users who want to utilize the Quartet quality control system. This is especially challenging for visualizing the genomics and transcriptomics data in multiple dimensions. In this regard, interactive visualization tools were developed to assist users in quickly exploring the reference datasets.

**Fig. 4:**
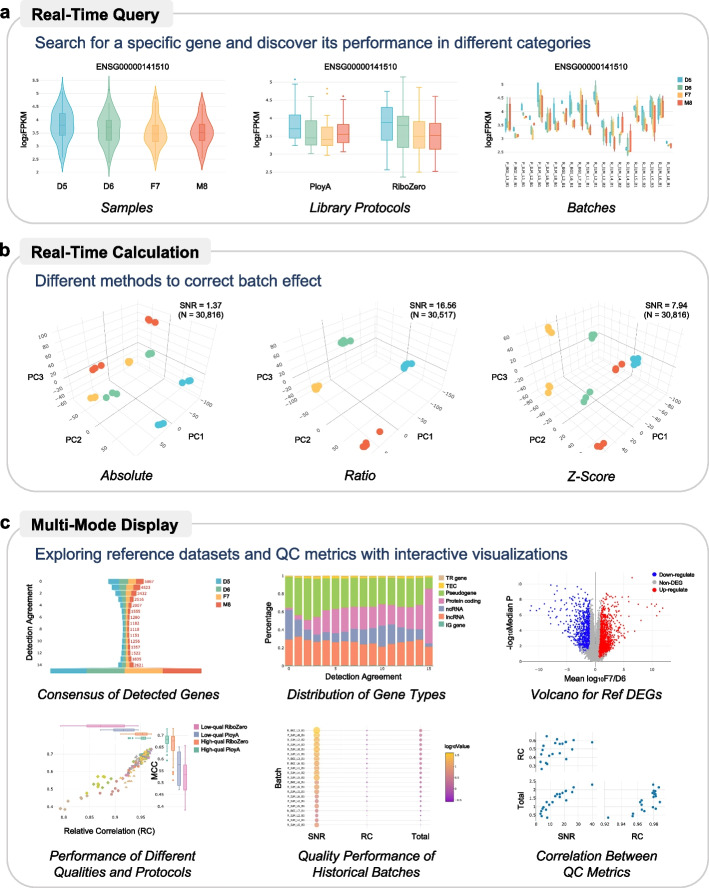
Online interactive visualization for instant exploration of the reference datasets. **a** Real-time query to obtain the expression status of a specific gene. **b** Real-time calculations are performed when different pre-processing methods are selected. **c** Multi-mode display approach helps users explore reference datasets from multiple perspectives

It features the following three functionalities. The first function is to perform a real-time query for the expression of specific genes, proteins, and metabolites (Fig. 4a). This function enables users to retrieve the expression level of the query objects under different conditions, e.g., samples, labs, protocols, and instruments. The second function is to integrate pre-processing methods for real-time calculation and visualization (Fig. 4b). This function allows users to select different batch combinations and corresponding methods for the correction of batch effects. The Principal Component Analysis (PCA) figures plotted in real-time can help the user choose the most appropriate ones. Finally, it allows the users to select the visualization module around the perspective that QC metrics focus on. As shown in Fig. 4c, which is a partial example of the visualization module for transcriptomics, users can understand the reference dataset better from different perspectives by using different display of interactive visualizations.

### Standardized quality assessment for user-submitted data

Online quality assessment has been built as part of applications in the Quartet Project quality control system which require account control. As previously mentioned, users with free access to reference materials must upload their in-house omics data generated with the Quartet reference samples and associated metadata in a timely manner. To facilitate data submission, the Quartet Data Portal leverages the Alibaba Cloud Object Storage Service (OSS) as the storage solution. Users can directly upload data from reference samples to the Quartet Data Portal using official tools such as the OSS utility and OSS browser. A detailed tutorial is accessible at https://docs.chinese-quartet.org/getting_started/submit_data/. The submitted data will be checked manually as well as by the metadata specification validation tool (https://github.com/chinese-quartet/metadata-validator) to ensure fidelity.

**Fig. 5:**
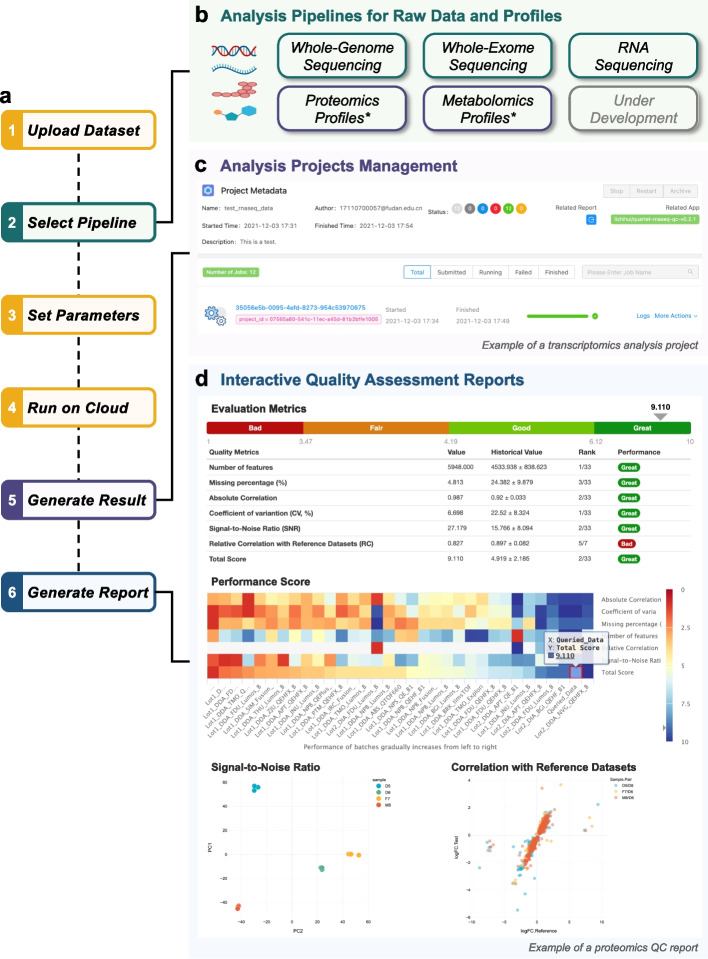
Quality assessment for user-submitted omic data. **a** A typical workflow for quality assessment of omic data on the Quartet Data Portal. **b** Analytical functionalities are organized as reproducible pipelines for whole-genome sequencing, whole-exome sequencing, RNA sequencing, proteomics profiles, and metabolomics profiles. **c** Historical analysis is managed on a project-by-project basis. Users can monitor the progress of tasks and manage the analysis results. **d** Interface of creating an quality assessment report and the generated interactive report

Following successful completion of these verification procedures, users are empowered to proceed with their own data analysis, i.e., selecting the specific pipeline and parameters to obtain the analysis results and QC results (Fig. 5a). Currently, the data portal offers bioinformatic analysis pipelines catering to various types of omics data, including whole-genome sequencing (WGS), whole-exome sequencing (WES), RNA sequencing (RNAseq), proteomics profiles, and metabolomics profiles (Fig. 5b). All analysis tasks are managed as individual projects within user accounts, enabling users to monitor the progress of their analysis and access related results for each sample (Fig. 5c).

The Quartet Data Portal provides support for the analysis of fastq files (level 1) from WGS, WES, and RNAseq. To minimize the potential influence of the analysis pipelines and facilitate an objective performance assessment from sample processing to data generation, the pipelines used are aligned with those employed in building the reference datasets [26, 27]. These pipelines are encapsulated using the Workflow Definition Language (WDL) and docker containers for seamless reproducibility [33–35]. Specifically, the pipelines for WGS and WES adhere to the best practices recommended by the Genome Analysis Toolkit (GATK), implemented using the Sentieon Genomics tools, which excels in the precisionFDA variant detection challenge [24]. For RNAseq, the read alignment and quantification processes involve a combination of HISAT, SAMtools, StringTie, and Ballgown [36]. The generated variant call files (VCF) as well as gene expression profiles are further used to calculate QC metrics to complete the performance assessment.

For proteomics and metabolomics, the development of full-flow applications starting from raw data is limited by the mass spectrometry technology itself [18]. Unlike transcriptomics, the subsequent analysis of the raw mass spectrometry data is platform-dependent, which is reflected in different parsing software, annotation databases, etc. In this case, we provide QC tools to support quality assessment from the quantification profiles (levels) onwards, i.e., directly starting from step 6 of Fig. 5a.

The last and most important step is the generation of the quality assessment report (Fig. 5d). This step provides an overall evaluation based on ranking quartiles, which are classified into four distinct categories, i.e., great, good, fair, and bad. Users conducting analysis on genomic or transcriptomic data have the convenience of directly selecting the corresponding project ID, while users who analyze proteomic or metabolomic data are required to upload profiles and set parameters. During this phase, the submitted data is evaluated based on the QC metrics specific to the corresponding omics discipline. The ranking of the submitted dataset will be provided by comparing its performance with that of representative historical batches integrated in the Quartet Project, where a higher score indicates a better ranking or quality. Consequently, performance levels are assigned based on predefined ranking ranges. The top 20% is recognized as “great”, the range between the top 20% and median 50% is labeled as “good”, the range between the median 50% and bottom 20% is denoted as “fair”, and the bottom 20% is categorized as “bad”. The calculation of total performance scores for each omics discipline is outlined as follows.

#### Qualitative omics: genomics

To provide an overall assessment, the total score was determined as the mean values of precision, recall, and mendelian concordance rate (MCR), where each component corresponds to an F0.5-measure combining the SNV score and the Indel score. Precision and recall assess the accuracy of variants within benchmark regions by comparing them with the benchmark variants. Precision represents the fraction of called variants in the test dataset that are true, while recall represents the fraction of true variants that are called in the test dataset. MCR is defined as the number of variants adhering to Mendelian inheritance laws divided by the total number of variants called within the Quartet samples. Mendelian concordant variants are those shared by the twins (D5 and D6) and exhibit Mendelian inheritance patterns with their parents (F7 and M8).

#### Quantitative omics: transcriptomics, proteomics, and metabolomics

Two pivotal QC metrics developed within the Quartet Project serve as vital indicators of quantitative omics data quality. Signal-to-noise ratio (SNR), which is reference dataset-independent, aims to assess the capability to discern intrinsic differences among distinct biological groups (“signal”) from variations arising from technical replicates within the same group (“noise”). A high SNR indicates the tight clustering of technical and wide dispersion of different samples groups, which characterizes good reproducibility and discriminability at the batch level. Relative correlation with reference datasets (RC), which is reference dataset-dependent, is employed to evaluate the quantitative consistency of user-submitted data with reference datasets at relative levels. The reference datasets, derived from high-quality historical datasets, serve as benchmarks for relative abundance values pertaining to each sample pair (D5/D6, F7/D6, and M8/D6). By calculating the relative values (ratios to D6) for the queried data, specifically for features that overlap with the reference datasets, the RC value is obtained as the Pearson correlation coefficient between the queried dataset and the reference dataset.

In addition to these two fundamental metrics, the calculation of the total score incorporates several omics-specific metrics. In the case of transcriptomics data, the total score was expressed as the product of SNR and RC. For proteomics data, the total score is the geometric mean of scaled values corresponding to the number of features, missing percentage, absolute correlation, coefficient of variation of technical replicates, SNR, and RC. As for metabolomics data, the total score is determined as the geometric mean of the recall of differential abundant metabolites (DAMs) in reference datasets, SNR, and RC. This recall metric serves as a qualitative assessment of the accuracy in detecting biological differences and represents the fraction of DAMs in the reference datasets that are successfully retrieved. Recall is calculated as the number of measured DAMs (*p* < 0.05, *t*-test) divided by the total number of DAMs that should be identified as part of the reference datasets.

### Application scenarios implemented with the Quartet Data Portal

**Fig. 6:**
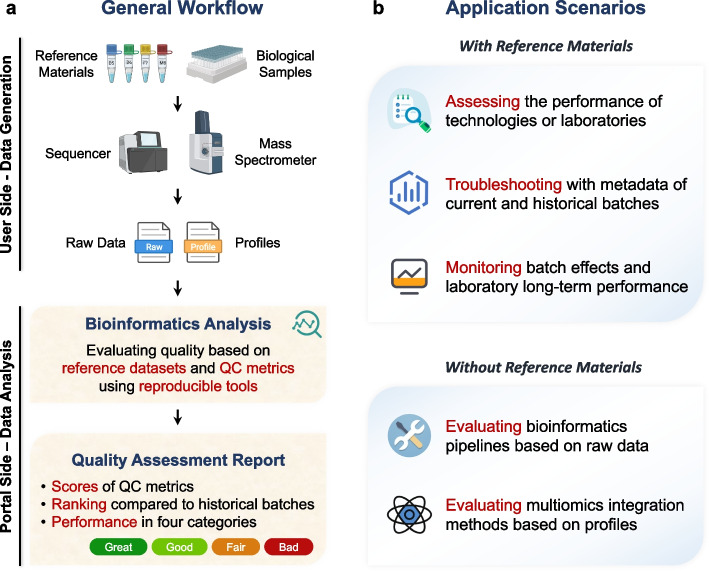
Application scenarios of the Quartet Data Portal. **a** General workflow of the main applications. The user performs experiments on reference samples and biological samples within the same batch. And after generating sequencing or mass spectrometry data, the user submits the resulting data to the Quartet Data Portal. The system will run a reproducible processing procedure to generate a comprehensive quality assessment report for the user. **b** Application scenarios with or without reference materials

A general application process involves the first three steps in the closed-loop process mentioned earlier (Fig. 6a). Initially, users request the Quartet reference materials and then perform experiments either individually or in batches alongside biological study samples. Subsequently, raw sequencing data or quantification profiles from the reference materials can be submitted to the portal for quality assessment, employing the online analysis tools. Finally, the submitted data will be subjected to scoring, ranking, and assigned an overall performance category based on the established best practices of Quartet Project.

Following the above process, users can achieve the outcomes as shown in Fig. 6b. First, the users can use the Quartet reference materials to evaluate the quality of their inhouse data generation steps. In particular, reference materials can play an important role in assessing the performance of new technology platforms and reagents, or the qualifications of service labs or operators. Secondly, in the multi-batch experiments, reference materials from each batch can be used to monitor batch effects and long-term lab performance. Besides, users can correct the batch effects based on the relative expression of Quartet samples (detailed in accompanying articles [25, 27, 30]). Thirdly, metadata of historical batches of the Quartet Project is helpful for troubleshooting. Sequencing and mass spectrometry experiments are complex and composed of multiple steps, but metadata records detailed experimental information about every step. Influencing factors can be eliminated case by case by comparing with other studies.

In addition, other users who have not applied for the Quartet reference materials can also make more in-depth use of the resources provided by Quartet Data Portal. Here we provide two scenarios. On the one hand, large amounts of raw data of the Quartet Project can be used to evaluate the performance of various bioinformatics pipelines. Specifically, the same sequencing data can be analyzed by using different pipelines and their performance can be evaluated based on the comparison of the obtained profiles with the reference datasets. On the other hand, multiomics profiles and the Quartet study design with built-in biological truth can be used to explore multiomics integration issues, e.g., performance evaluation of integration tools.

## Discussion

The Quartet Data Portal integrates the unique resources and results from the Quartet Project with various tools, e.g., interactive visualization and online quality assessment, to facilitate the application of the quality control system based on the Quartet reference materials by the community. Currently, the portal involves multiple steps such as data storage, data analysis, and quality report. Guided by the FAIR principles [31], multi-level data related to the Quartet Project are managed and published uniformly by the platform in a version-controlled fashion. To date, multiple studies based on data published by the Quartet Data Portal have been successfully conducted [37, 38].

Best practices built in the Quartet Project for the quality assessment of sequencing data and mass spectrometry profiles have been provided as online reproducible analysis tools in the Quartet Data Portal. These tools have been intentionally selected to align with the methodologies employed in constructing the reference datasets discussed in the accompanying studies [26, 27]. It is recognized that relying on a single analysis pipeline may introduce biases in variant detection and gene expression quantification. To enhance the rigor of the reference datasets, further comprehensive evaluations and the incorporation of additional bioinformatics tools are planned for subsequent updates [39, 40]. Furthermore, the direct analysis of raw datasets to generate quantification profiles for proteomics and metabolomics is currently not supported by the platform. Presently, users are limited to uploading profiling results in the designated format. However, there are plans to enhance the QC tools for proteomic and metabolomic analysis in the future. This improvement will be facilitated by leveraging a larger pool of community data and incorporating more advanced analysis software.

With the widespread use of the Quartet reference materials by the community, we will inevitably face the challenge of integrating data from different sources and the continuous evolution of the QC system integrated in the Quartet Data Portal [41]. The importance of reproducible data analysis pipelines and structured metadata formats for the community-wide multiomics research is well-acknowledged [42–44]. Consequently, we have built this Portal to address these unmet needs. In terms of metadata specification, we have structured the metadata of the whole experiment and analysis processes and defined the fields with the knowledge of ontology. This helps users interpret the data correctly and also makes comparisons with other studies more straightforward and meaningful. To ensure the computational reproducibility, analysis pipelines are strictly versioned, and the software within the pipelines are packaged using docker containers [33–35]. The QC tools that users can choose from are also consistent with the version of the reference datasets and QC metrics.

The “distribution-collection-evaluation-integration” model implemented in the portal allows more researchers to be truly involved in the quality control of multiomics studies. However, there are still some challenges that need to be addressed step-by-step in the future. First, the current release of multi-level datasets requires the manual collation by the Quartet team on a regular basis. In the future, we plan to develop automated execution processes as well as more granular provisions to avoid the potential delay and bias of manual processing. In addition, a wider variety of reproducible pipelines and analysis modules (e.g., local standalone software) are envisioned to help researchers perform more personalized analyses.

In summary, we have made an attempt in promoting the multiomics research community to work together to solve quality problems. Our intention is to integrate community-generated data while sharing the public with Quartet reference materials. The “distribution-collection-evaluation-integration” closed-loop model drives the evolution of reference datasets, QC metrics, and QC tools for small variants, structural variants, mRNAs, proteins, and metabolites. We believe that the Quartet Data Portal can be useful for multiomics studies, helping raise awareness of quality control among researchers in the community and laying a solid foundation for more reliable biological discoveries.

## Conclusions

The Quartet Data Portal represents a comprehensive platform that actively involves the research community in the convenient utilization and enhancement of Quartet resources. The Quartet datasets derived from the genomics, transcriptomics, proteomics, and metabolomics reference materials provide a comprehensive overview of data quality across different omics profiling. The integration of functionalities for requesting reference materials, interactive visualization tools and reproducible analysis tools fosters effective interaction and mutual benefit between the research community and the Quartet Project. These collaborative endeavors hold significant promise in enabling the research community to advance quality control and standardization of multiomics research practices.

## Methods

The construction of the Quartet Data Portal consists of four main parts: multiomics data management module, data analysis and quality assessment module, quality report module, and visualization dashboard module.

### Multiomics data management module

The Quartet Data Portal has a set of solutions that support flexible customization and expansion of metadata, and can reflect the structural relationship between metadata and support version upgrades and evolution. The current metadata solution includes project, donor, sample, reference material, library, sequencing, datafile and other entity information to track the details of the entire process of data generation and analysis. This module relies on the NoSQL databases including MongoDB and Nebula Graph DB, to handle the storage of a large amounts of semi-structured data, and object storage, i.e., S3, MinIO, Alibaba Cloud Object Storage Service (OSS), to realize the storage of a large number of omics data files.

The verification of the Quartet metadata is a key step for effective data management. All metadata is included in the unified management system and needs to follow strict data types, limit value ranges, and check for validity of values, etc., while supporting metadata model extension and version replacement. Therefore, we have defined a set of “Data Package” specifications to implement the constraints and verification of metadata. It is mainly composed of a set of specific directory structures and several CSV files. Each CSV file corresponds to a model description file based on JSON schema to complete the verification of the corresponding data structure and contents.

### Data analysis and quality assessment module

Multiomics data usually require a relatively large storage and computation capacities. For quality control of the raw data, computing resources are required, computing time is long, and quality control pipelines related to different omics data are different. The Quartet Data Portal requires a set of computing systems dedicated to multiomics data analysis and flexible definition of quality control pipelines to build quality assessment modules. Therefore, we have defined a set of specifications to realize the encapsulation and definition of the pipelines, which is mainly based on Workflow Definition Language (WDL), template language and catalog specifications. In order to meet the needs of cross-platform scheduling computing tasks, we implemented a computing system that supports custom application specifications based on the Cromwell scheduling engine. It is combined with a series of omics data quality control applications to complete the quality assessment of multiomics data.

### Quality report module

There are many quality evaluation indicators, and the content of the quality control report of different types of omic data is different. The Quartet Data Portal requires a set of quality assessment report modules that support custom report contents and styles and can be used to interactively explore results. Therefore, we use Clojure language to implement a set of quality assessment report modules that support plug-in mechanism, and all report plug-ins are built based on MultiQC [45] and Plotly (https://github.com/plotly/plotly.py). Report plug-ins can be added and deleted flexibly to complete the generation and display of corresponding quality assessment reports.

### Visualization dashboard module

The visualization dashboard is developed in the R language (version v3.5.1) (https://www.r-project.org). The shiny v1.2.0 and shinydashboard v0.7.1 are used to deploy on a self-managed webserver based on the Shiny Server (https://www.rstudio.com/products/shiny/shiny-server/). The widgets of the webserver are developed by shinyWidgets v0.4.8. The analysis tables are processed by data.table v1.12.8 and feather v0.3.5 due to their high efficiency, and data are manipulated using the plyr v1.8.4, dplyr v1.0.2 and purrr v0.3.2. The visualization module is developed using the R package plotly v4.9.2.1, which includes scatter, box, bar, violin, heatmap, funnel and splom types. Except for the heatmap and dendrogram, the R package heatmaply v1.1.1 is used. The other section of the genome visualization module uses R package plotly v4.9.2.1 and ggplot2 v3.3.2, which includes scatter, line, box and bar types. The theme of dashboard is developed through dashboardthemes v1.1.3, and the theme of ggplot2 is mainly through ggthemes v4.2.0. The colors of the plots are set using RcolorBrewer v1.1–2. The data values are mapped to the graph using lattice v0.20–35 and scales v1.1.1. Specifically, the renv v0.11.0 R package is invoked to bring project-local R dependency management to the project.

### Supplementary Information

**Additional file 1.** Review history

## Data Availability

The multiomics data are available at the Quartet Data Portal and the National Genomics Data Center of China with BioProject ID of PRJCA012423 [46]. The raw data of WGS, WGBS, RNAseq and miRNAseq are deposited in the Genome Sequence Archive (GSA) [47] (accession number: HRA001859) [48]. The Illumina EPIC array data are deposited in the Gene Expression Omnibus (GEO) [49] (accession number: GSE241900) [50]. The mass spectrometry proteomics data are deposited in the ProteomeXchange Consortium via the iProX partner repository [51, 52] (accession numbers: PXD043262 and PXD045065) [53, 54]. Variant calling files are deposited in the European Nucleotide Archive (ENA) [55] (accession number: PRJEB66342) [56]. RNAseq, miRNAseq and metabolomics profiles were deposited in figshare [57, 58]. The source codes for quality assessment based on Quartet multi-omics reference materials and reference datasets are available on GitHub under either MIT or EPL-2.0 licenses [59] and Zenodo [60–64].
