# Supplementary material for: The Quartet Data Portal: integration of community-wide resources for multiomics quality control
Source: Genome Biol. 2023 Oct 26;24:245. doi: 10.1186/s13059-023-03091-9 (PMC10601216; doi:10.1186/s13059-023-03091-9)
Supplement: Supplementary file 1 — Additional file 1. Review history [file 13059_2023_3091_MOESM1_ESM.docx]

Review History

**First round of review**

**Reviewer 1**

**Were you able to assess all statistics in the manuscript, including the appropriateness of statistical tests used?**

There are no statistics in the manuscript.

**Have you been able to install and test the software?**

No

**Comments to author:**

Major comments:


1) Terminology needs to be clarified. Can author clearly define what is reference materials? Are those fastq file? Physical samples etc
2) It was not clear how the performance metrics and pipeline will be decided? Will that be based on some sort of benchmarking
3) Section background line 52. It is not clear what authors mean by recourses
4) Section background. It would be helpful to illustrate what authors mean by reference materials on example of GIB
5) Line 55. It would be helpful to provide overview of each of the data portals and say what types of information do the provide including reference materials
6) Line 79. Manuscript sare not yet published, so it better to not refer them as papers instead could be feasible to refer to them as preprints
7) Function of the portal needs to clearly defined - for example can user request all samples and if there is any restrictions based on the country. Also what will be the associated cost with it. If so, how the money received will be spend and who will decide that.
8) Is the omics data under the portal can be used freely? If so this needs to be emphasized. Current language includes "public to request". Does it refer only to physical samples? Or to data as well?
9) Any reason BAM files are not shared and only FASTQ files are shared?
10) One severe limitation of the proposed data portal as compared to the efforts like GIB is lack of gold standards. There are multiple types of gold standards (see https://www.nature.com/articles/s41467-019-09406-4 ) . Such limitation needs to be discussed
11) How long is the current approval time?
12) The model behind the data portal needs to be described with more details. For example, the manuscripts states that users will be encouraged to upload raw data, can it be made a requirement to share data publicly both at he described data portal as well as at SRA/GEO
13) Line 196. It will be beneficial for the data portal to adopt more stringent requirements - for example - user generated multii omics data MUST be submitted for real time quality check not can be … and shared on publicly available repositories (e.g. SRA, GEO )
14) It is not clear how tools for analysis are selected. The choice needs to be a result of rigorous benchmarking . See https://www.nature.com/articles/s41467-019-09406-4 and https://www.biorxiv.org/content/10.1101/2022.09.22.508982v1


This needs to be discussed as one of the limitation of the data portal
15) Line 231. I am not sure why 2) and 3) considered as challenge
16) Line 284. It will be beneficial to cite a review about containerization of bioinformatics software. For example , https://arxiv.org/abs/2203.16261
17) Fig 2b. How # tybes is related to number of samples. It would be helpful to have a similar figure but for number of samples
18) Fig 2. Multi sites profiling was not defined
19) It would be helpful for reader to know how many samples were sequenced using the same technology in different laboratories and how many samples were sequenced with different technologies (for example with Illumina and some other technology). This information can be visualized and added to the Fig 2
20) Is there is a strategy developed to make sure completeness of metadata accompanying omics data. Specially for the omics data uploaded by the users. It was recently demonstrated that metadata is substantially incomplete. For example,
21)


Minor comments

1) In abstract it is mentioned - "user submitted multi omics data". I am not sure if the users will always submit multiple or single omics data
2) It would be helpful to cite literature on reproducibility in biomedical research - for example https://academic.oup.com/gigascience/article/9/6/giaa056/5849489
3) Line 135 It will be helpful to provide full names of MGI and DNB
4) Line 231. It is better to avoid to avoid 1) 2) in the text instead use plain English

**Reviewer 2**

**Were you able to assess all statistics in the manuscript, including the appropriateness of statistical tests used?**

There are no statistics in the manuscript.

**Have you been able to install and test the software?**

Yes

**Comments to author:**

In this manuscript, the authors present the Quartet data portal, a web site dedicated to the Quartet project.
The web site offers 4 main functionalities: material request, data download, QC, and reference data.
The samples can be ordered to be used in new Omics experiments, the portal data can be downloaded, a user's data can be analyzed, and the reference analysis can be explored.
It supports multiple types of omics data: DNA, RNA, Proteomics and Metabolomics.
Although the data portal looks promising, its scope is quite narrow and its visualizations urgently need annotations to be understood.

Major comments:

The manuscript must say clearly that that the all portal and potential future data are about the biological samples from 4 individuals of the same family only. If I did not understand correctly, please update the manuscript for clarity.
Online plots can not be understood : mostly short acronyms, no legend, no idea about the experiments being summarized (boxplots out of what datasets?)
Some online pages also suffer from lack of documentation. As an example among others: the user has no idea about the metadata of the samples or experiemtns to be downloaded.
Manuscript plots must be understable by the reader : all parts must be made clear
Manuscript plots are often not readable on a letter- or A4-sized sheet of paper : text is often too small
Manuscript plots have poor legend that do not help to understand the multiple acronyms and the meaning of some visualization (e.g. bar size)
The reader has little idea about the relevance of the QC analyses offered by the data portal: manuscript should include detailed description of the QC analyses, tools, decision thresholds or ranking of samples or datasets

Minor comments:

The manuscript must say clearly that login is required for some functionalities
"After approval of the Quartet team": please elaborate and write clearly any excluding criteria

**Authors Response**

**Point-by-point responses to the reviewers’ comments:**

**Reviewer #1:**

**Major comments:**

1) Terminology needs to be clarified. Can author clearly define what is reference materials? Are those fastq file? Physical samples etc

**Reply:** We sincerely appreciate your invaluable suggestions. In this manuscript, the term “reference materials” means the physical samples possessing known properties. It is noteworthy that the Quartet Data Portal has been developed to facilitate community access to well-characterized reference materials, reference datasets, and related resources from the Quartet project. The Quartet multiomics reference materials encompass DNA, RNA, Protein, and metabolite samples that align with the definition of reference materials as outlined in ISO Guide 30:2015. These materials exhibit the necessary homogeneity and well-established property values essential for instrument calibration, the assessment for measurement and computational methods. Additionally, it is worth mentioning that the reference materials for DNA and RNA within the project have attained the status of a certified reference material (CRM). These materials serve the purpose of validating the accuracy and dependability of analytical methodologies, evaluating data comparability across different laboratories or instruments, and establishing a benchmark for assessing the precision and accuracy of measurements. We have duly incorporated a comprehensive definition of reference materials and more detailed background within the revised manuscript, specifically in lines 45-47.

2) It was not clear how the performance metrics and pipeline will be decided? Will that be based on some sort of benchmarking

**Reply:** Thank you for your question. The performance metrics and pipelines in Quartet Data Portal are established by the Quartet Project, which has been a huge undertaking over the past seven years of several dozens of organizations within MAQC China and beyond. In this manuscript, we are delighted to develop a platform for the scientific community to conveniently access the Quartet multiomics resources. Four accompanying manuscripts have been prepared, outlining the establishment of reference materials, reference datasets, and quality control (QC) methods for DNA (Ren L et al., *bioRxiv*, 2022), RNA (Yu Y et al., *Nature Biotech.*, 2023, Accepted), protein (Tian S et al., *bioRxiv*, 2022),

and metabolite (Zhang N et al., *bioRxiv*, 2022) analyses. Comprehensive information on the determination of performance metrics and pipelines can be found in the corresponding manuscripts. In the revised manuscript, we also added a detailed explanation of the analysis pipelines and QC metrics employed for each omics type in lines 271-346.

3) Section background line 52. It is not clear what authors mean by recourses

**Reply:** We appreciate your question. The term “resources” in our study are materials and tools that can be used for quality control of multiomics profiling, whose definitions have been added to lines 48-54 of the manuscript. These include Quartet multiomics reference materials, diverse datasets that have been derived from these materials, high-confidence reference datasets accompanied by quality control metrics, as well as reproducible analysis pipelines for implementing best practices.

4) Section background. It would be helpful to illustrate what authors mean by reference materials on example of GIB

**Reply:** Thank you very much for your suggestion. We have added the GIAB website as an example of an existing website that does not fully meet the needs of users seeking an integrated workflow from sample acquisition to data quality assessment. Please see lines 61-67 of the revised manuscript for details.

5) Line 55. It would be helpful to provide overview of each of the data portals and say what types of information do the provide including reference materials

**Reply:** We appreciate your valuable advice. The overview of data portals for quality control studies based on reference materials has been added in **Table 1**.

6) Line 79. Manuscript sare not yet published, so it better to not refer them as papers instead could be feasible to refer to them as preprints

**Reply:** Thank you for your suggestion, it has been changed to “preprints” in our revised manuscript.

7) Function of the portal needs to clearly defined - for example can user request all samples and if there is any restrictions based on the country. Also what will be the associated cost with it. If so, how the money received will be spend and who will decide that.

**Reply:** Thank you for your valuable suggestions. As you mentioned, it is crucial for prospective users of Quartet reference materials to have a clear understanding of the fundamental principles, constraints, and associated costs involved in acquiring the Quartet reference materials. In response to this concern, we have incorporated a detailed description of the reference materials acquisition policy within the documentation system (<https://docs.chinese-quartet.org/policies/reference_materials_policy/).>

In summary, global users are eligible to apply for free access to the reference materials strictly for research purposes, with shipping costs borne by the users themselves. For any commercial use of the reference materials, users are required to contact the Quartet team for further communication. The following applications will be declined. The Quartet team will strive to provide explicit reasons for the rejection and offer constructive suggestions to assist applicants in obtaining the reference materials smoothly and promptly.

- Incomplete or inaccurate applications lacking essential information.
- Non-compliant applications that violate the national laws, regulations, or policies.
- Unreasonable quantities of requested reference materials.
- Commercial purposes without agreements with administrators.
- Refusal to cover necessary costs, i.e., shipping fees.
- Refusal to provide feedback on the utilization and analysis results.

8) Is the omics data under the portal can be used freely? If so this needs to be emphasized. Current language includes "public to request". Does it refer only to physical samples? Or to data as well?

**Reply:** Thank you for your question. The term “public to request” refers to both physical samples and data. The multiomics datasets released on the Quartet Data Portal are open-access, which has been approved by HGRAC. Please refer to the previous response for cases where free access to reference materials is not supported. We have added the permissions policy of the Quartet Data Portal in lines 193-228 of the revised manuscript.

9) Any reason BAM files are not shared and only FASTQ files are shared?

**Reply:** Thank you for your review. The Quartet Project has been a huge undertaking over the past seven years of several dozens of organizations within MAQC China and beyond in pursuit of quality control and data integration of multiomics profiling. Given the extensive duration and the involvement of multiple organizations in the analysis, not all intermediate data from every omics domain have been retained. Nonetheless, it is crucial to highlight that the raw data and metadata of utmost significance have been meticulously preserved and published. Moving forward, as part of our ongoing system updates, we will consider reanalyzing historical data and implementing proper measures to preserve each level of data comprehensively.

0) One severe limitation of the proposed data portal as compared to the efforts like GIB is lack of gold standards. There are multiple types of gold standards (see <https://www.nature.com/articles/s41467-019-09406-4)>. Such limitation needs to be discussed

**Reply:** We appreciate your insights on our study. In response to your comment, we would like to clarify the nature of the Quartet Data Portal as an application platform for accessing and using the “gold standard” developed in Quartet Project. In our study, the term “gold standard” refers to the “high-confidence reference datasets”, which align with the definition provided by the Genome in a Bottle Consortium (GIAB). Reference datasets were built using the consensus voting principle, based on the high-quality datasets generated across omics, platforms, labs, protocols, and batches. In the field of genomics, benchmark calls encompass 4.2 million small variants and 15,000 structural variants for evaluating the reliability of germline variant calls inside the high-confidence regions (Ren L, et al. *bioRxiv*, 2022). For quantitative omics, ratio-based reference datasets and quality metrics were established to assess reliability in terms of intra-batch proficiency and cross-batch reproducibility (Yu Y, et al. *Nature Biotech.*, 2023, Accepted; Tian S, et al. *bioRxiv*, 2022; Zhang N, et al. *bioRxiv*, 2022). Thank you once again for your valuable comments to our study.

1) How long is the current approval time?

**Reply:** Thank you for your question. We can process and evaluate users’ requests for reference materials and/or account applications within 3-7 working days.

2) The model behind the data portal needs to be described with more details. For example, the manuscripts states that users will be encouraged to upload raw data, can it be made a requirement to share data publicly both at he described data portal as well as at SRA/GEO

**Reply:** We highly value your insightful suggestion. The guidelines regarding data uploads have been elucidated in lines 220-228. We impose mandatory data submission for users with free access to reference material and require their consent to open access to the public through GSA, SRA, etc. To facilitate this process, we provide research users with an electronic version of the Data Authorization and Open Access Agreement prior to providing them with the reference materials. Additionally, we offer metadata specifications and complementary validation tools to ensure the integrity and consistency of the shared data (available at <https://github.com/chinese-quartet/metadata-validator).>

In accordance with the Reference Material Request Policy, research users who decline to share their data will either be ineligible to re-access reference materials or may be required to transition to commercial user status. Further details can be found at <https://docs.chinese-quartet.org/policies/data_submission_policy/.>

3) Line 196. It will be beneficial for the data portal to adopt more stringent requirements - for example - user generated multii omics data MUST be submitted for real time quality check not can be ... and shared on publicly available repositories (e.g. SRA, GEO)

**Reply:** We fully endorse your suggestion. As previously mentioned, our objective is to enforce real-time quality checks through the implementation of the Data Authorization and Open Access Agreement with our users. The Quartet team will carry out regular curations to ensure the data integrity and then upload it to publicly available repositories in a uniform manner. The associated data is concurrently hosted in both NODE and GSA, and we are currently considering expanding its availability to more public repositories such as SRA and ENA. The data policy for the Quartet Data Portal is now integrated into the documentation website, which can be accessed at <https://docs.chinese-quartet.org/policies/data_request_policy/.>

4) It is not clear how tools for analysis are selected. The choice needs to be a result of rigorous benchmarking. See <https://www.nature.com/articles/s41467-019-09406-4> and <https://www.biorxiv.org/content/10.1101/2022.09.22.508982v1>. This needs to be discussed as one of the limitation of the data portal

**Reply:** Thank you for your valuable suggestion. It is my understanding that the term “tools” mentioned in your comment should refer to the data processing steps involved in transforming raw genomic or transcriptomic data into mutation or expression profiles. These tools utilized on our website were deliberately chosen to align with those employed in constructing the reference dataset, which are validated in the accompanying studies (Ren L et al., *bioRxiv*, 2022; Yu Y et al., *Nature Biotech.*, 2023, Accepted). This decision was made to minimize the potential influence of the analysis pipelines and facilitate an objective assessment of proficiency testing throughout the wet experiment, spanning from sample processing to data generation.

Moreover, it is worth noting that the accompanying studies also exercised great care in the selection of appropriate analysis pipelines and objectively discussed the potential biases introduced by relying on a single pipeline. The analysis pipelines employed for whole-genome sequencing (WGS) and whole-exome sequencing (WES) adhered to the best practices recommended by the Genome Analysis Toolkit (GATK), utilizing the Sentieon genomics tools. Notably, the latter demonstrated exceptional performance in the variant detection challenge conducted by precisionFDA, thereby substantiating its reliability and effectiveness for variant analysis. Regarding RNA sequencing (RNAseq), the read alignment and quantification processes were conducted using a combination of HISAT, SAMtools, StringTie, and Ballgown. These methods were compared to the Bowtie2 and RSEM processes. Notably, the differences observed between the two approaches were minimal, particularly when evaluating the quality assessment at the ratio level.

Considering the aforementioned factors, we did not explicitly delve into the specific details of benchmarking within this paper. Nevertheless, we wholeheartedly concur with your suggestion to provide further clarification on this matter within the discussion section. We sincerely appreciate your valuable feedback, as it plays a crucial role in enhancing the scientific rigor and quality of our manuscript.

5) Line 231. I am not sure why 2) and 3) considered as challenge

**Reply:** We appreciate your question and would like to provide clarification on the matter. In our manuscript, the references to 2) and 3) in line 231 (now line 352) refer to the second and third steps within the closed-loop process of the Quartet Data Portal's interaction with the scientific community, rather than specifically addressing the challenge itself. These steps represent integral components of the Quartet Data Portal's engagement model, designed to facilitate effective collaboration and knowledge exchange between researchers. Step 2 involves the active participation of users within the community, enabling them to contribute their own datasets, analyses, and insights to the platform. Step 3 encompasses the evaluation and validation of the contributed data and analyses through rigorous quality control measures and benchmarking against reference standards. It is essential to note that these steps are part of the broader framework of the Quartet Data Portal's functionality, facilitating a dynamic and iterative process of interaction and collaboration within the scientific community. We hope this clarification addresses your query. Thank you for your valuable contribution to the peer review process, which helps us enhance the clarity and comprehensiveness of our manuscript.

6) Line 284. It will be beneficial to cite a review about containerization of bioinformatics software. For example, <https://arxiv.org/abs/2203.16261>

**Reply:** Thanks for your valuable advice. We have added this reference to the manuscript.

7) Fig 2b. How # tybes is related to number of samples. It would be helpful to have a similar figure but for number of samples

**Reply:** Thanks for your question. To provide a more accurate understanding of the total number of preparations, we have included detailed information on the specifications for each omics in lines 179-181. In addition, we have added this figure (i.e., **Fig. 2c**) with the classification of samples as x-axis, and different colors indicate different omics types.

8) Fig 2. Multi sites profiling was not defined

**Reply:** Thank you very much for your review. We appreciate your attention to detail and have made the necessary correction in **Fig. 2a**, where the term “site” has been replaced with “lab” to accurately represent the experimental setting.

9) It would be helpful for reader to know how many samples were sequenced using the same technology in different laboratories and how many samples were sequenced with different technologies (for example with Illumina and some other technology). This information can be visualized and added to the Fig 2

**Reply:** Thank you very much for your suggestions. We have added a **Fig. 2b** for a more direct presentation of the information.

10) Is there is a strategy developed to make sure completeness of metadata accompanying omics data. Specially for the omics data uploaded by the users. It was recently demonstrated that metadata is substantially incomplete. For example,

**Reply:** We sincerely appreciate your inquiry. To ensure the accessibility and reliability of the data, our team has devised a metadata specification along with a complementary validation tool (<https://github.com/chinese-quartet/metadata-validator)>. Users are afforded the flexibility to conduct metadata validation either locally or through the Quartet Data Portal. The provided validation tool serves to simplify the creation of metadata template tables by offering guidance and examples for accurately completing metadata fields. It additionally verifies the format and content of the metadata, generating a comprehensive validation log that highlights any identified issues and offers recommendations for rectification.

**Minor comments**

1) In abstract it is mentioned - "user submitted multi omics data". I am not sure if the users will always submit multiple or single omics data

**Reply:** Thanks for your question. The Quartet Data Portal provides online QC for genomics, transcriptomics, proteomics and metabolomics data separately. Users are required to submit one type of omics dataset at a time. To avoid any ambiguity, we have removed the term "multiomics" from this sentence.

2) It would be helpful to cite literature on reproducibility in biomedical research - for example <https://academic.oup.com/gigascience/article/9/6/giaa056/5849489>

**Reply:** Thanks for your valuable advice. We have added this reference in the manuscript.

3) Line 135 It will be helpful to provide full names of MGI and DNB

**Reply:** Thank you very much for your careful review. It is important to note that “MGI” represents the official English name of the company and is not an abbreviation defined by us. Additionally, we would like to clarify that “DNB” stands for DNA nanoball, a technology employed in DNA sequencing. However, it is essential to recognize that “DNB” is an integral component of the product name and should be understood in a similar context as “Nova” is associated with “NovaSeq”.

4) Line 231. It is better to avoid to avoid 1) 2) in the text instead use plain English **Reply:** Thanks for your valuable advice. We have modified the format.

**Reviewer #2:**

In this manuscript, the authors present the Quartet data portal, a web site dedicated to the Quartet project.

The web site offers 4 main functionalities: material request, data download, QC, and reference data.

The samples can be ordered to be used in new Omics experiments, the portal data can be downloaded, a user's data can be analyzed, and the reference analysis can be explored.

It supports multiple types of omics data: DNA, RNA, Proteomics and Metabolomics.

Although the data portal looks promising, its scope is quite narrow and its visualizations urgently need annotations to be understood.

**Major comments:**

The manuscript must say clearly that that the all portal and potential future data are about the biological samples from 4 individuals of the same family only. If I did not understand correctly, please update the manuscript for clarity.

**Reply:** We appreciate your valuable suggestions. Yes, all data in the Quartet Data Portal are generated from the four individuals of the same family. We have made it clearly in the revised manuscript (lines 28-31).

Online plots can not be understood: mostly short acronyms, no legend, no idea about the experiments being summarized (boxplots out of what datasets?)

**Reply:** Thank you very much for your review. We have enhanced the online visualization module by incorporating experimental design information, metadata descriptions, and legends for the displayed figures. These additions aim to provide users with a comprehensive understanding of the data and facilitate their interpretation and analysis.

Some online pages also suffer from lack of documentation. As an example among others: the user has no idea about the metadata of the samples or experiemtns to be downloaded.

**Reply:** Thank you very much for your review. We have added data request guidelines in lines 203-211. A detailed tutorial on data download is accessible at <https://docs.chinese-quartet.org/getting_started/request_omics_data/.>

Manuscript plots must be understable by the reader: all parts must be made clear

Manuscript plots are often not readable on a letter- or A4-sized sheet of paper: text is often too small

Manuscript plots have poor legend that do not help to understand the multiple acronyms and the meaning of some visualization (e.g. bar size)

**Reply:** Thank you very much for your concern about the quality of the figures in this manuscript. Some of the figures had an issue with the font size being inappropriate as it was a visualization taken directly from the website. We have reworked the figure and legend, added annotations and enlarged the font.

The reader has little idea about the relevance of the QC analyses offered by the data portal: manuscript should include detailed description of the QC analyses, tools, decision thresholds or ranking of samples or datasets

**Reply:** Thank you for your question and suggestions. In the revised version of the manuscript, we provided a detailed explanation of the analysis pipelines and QC metrics employed for each omics in lines 271-346. More information on the determination of performance metrics and pipelines can be found in four accompanying manuscripts, outlining the establishment of reference materials, reference datasets, and quality control (QC) methods for DNA (Ren L et al., *bioRxiv*, 2022), RNA (Yu Y et al., *Nature Biotech.*, 2023, Accepted), protein (Tian S et al., *bioRxiv*, 2022), and metabolite (Zhang N et al., *bioRxiv*, 2022) analyses.

**Minor comments:**

The manuscript must say clearly that login is required for some functionalities

"After approval of the Quartet team": please elaborate and write clearly any excluding criteria

**Reply:** Thank you for your suggestions. We have added the permissions policy of the Quartet Data Portal in lines 193-228. In brief, access to Quartet reference material is free of charge for scientific research use and by agreeing to share the data with the Quartet team and for future public access through GSA, SRA, etc. Compliance with the regulations of the Human Genetic Resources Authority of China (HGRAC) is also required.

**Second round of review**

**Reviewer 1**

My comments were addressed

**Reviewer 2**

In this revised version, authors have addressed most of my comments.

Although still limited, the authors have added details about the methods and QC evaluation in the manuscript. Together with more details provided in their documentation online, it should be sufficient for users to understand the analysis.

Documentation of online plots have been improved. Some problems still exist such as in PCA plots where shapes (circles, triangles, ...) have no corresponding legend.

Some bugs also exist for example in PCA for group libraryPrep, it fails when selecting RiboZero as "Element of Group" together with PolyA. I encourage authors to test their web site / web app more carefully.


Minor comment:

L162: four short-read => four short-read sequencing platforms

**Authors Response**

**Point-by-point responses to the reviewers’ comments:**

We are delighted that both reviewers found our response satisfactory.

For the problem mentioned by Reviewer 2, we have corrected the bugs and added legends. The error message flashes in some scenarios only because the system is dynamically adjusting other options when switching between them. This error message is transient and does not affect the final result.
